# Complementary use of cardiac magnetic resonance and 18 F-FDG positron emission tomography imaging in suspected immune checkpoint inhibitor myocarditis

**DOI:** 10.1186/s40959-024-00250-0

**Published:** 2024-08-22

**Authors:** Jieli Tong, Nikolaos Vogiatzakis, Maria Sol Andres, Isabelle Senechal, Ahmed Badr, Sivatharshini Ramalingam, Stuart D. Rosen, Alexander R. Lyon, Muhummad Sohaib Nazir

**Affiliations:** 1 Cardio-Oncology Service, Royal Brompton Hospital, Guy’s and St. Thomas’ NHS Foundation Trust, London, UK; 2 School of Biomedical Engineering and Imaging Sciences, King’s College London, Guy’s and St Thomas’ Hospital, London, UK; 3 Department of Cardiology, Tan Tock Seng Hospital, Singapore, Singapore

**Keywords:** CMR, ^18^F-FDG PET, ICI myocarditis

## Abstract

**Background:**

Immune checkpoint inhibitor (ICI) myocarditis is an uncommon but potentially fatal complication of immunotherapy. Cardiac imaging is essential to make timely diagnoses as there are critical downstream implications for patients.

**Objective:**

To determine the agreement of cardiac magnetic resonance (CMR) and 18 F-fluorodeoxyglucose Positron Emission Tomography (FDG-PET) in patients with suspected ICI myocarditis.

**Methods:**

Patients with suspected ICI myocarditis, who underwent CMR and 18 F-FDG-PET imaging at a single cardio-oncology service from 2017 to 2023, were enrolled. CMR was performed according to recommended guidelines for assessment of myocarditis. 18 F-FDG-PET imaging was performed following 18 h carbohydrate-free fast. Imaging was analysed by independent reviewers to determine the presence or absence of ICI myocarditis.

**Results:**

Twelve patients (mean age 60 ± 15 years old, 7 [58%] male) underwent both CMR and 18 F-FDG-PET imaging. Three (25%) met the 2018 Lake Louise Criteria for CMR diagnosis of myocarditis; 4 (33%) had evidence of myocardial inflammation as determined by 18 F-FDG-PET. Amongst those with positive 18 F-FDG-PET, mean standard uptake value (SUV) was 3.5 ± 1.7. There was agreement between CMR and PET in 7 cases (CMR and PET positive (*n* = 1), CMR and PET negative (*n* = 6)) and discordance in 5 cases (CMR positive and PET negative (*n* = 2), CMR negative and PET positive (*n* = 3)).

**Conclusion:**

Both CMR and PET provide complementary clinical information in diagnostic of ICI myocarditis. CMR informs on myocardial oedema, whilst 18 F-FDG-PET provides information on glucose metabolism reflecting monocyte and lymphocytic activity. Future studies should investigate the role of hybrid PET-CMR for the timely diagnosis of ICI myocarditis.

**Supplementary Information:**

The online version contains supplementary material available at 10.1186/s40959-024-00250-0.

## Introduction

Immune checkpoint inhibitor (ICI) therapy has revolutionised the management of several cancers [1]. Despite the clinical effectiveness of these therapies, there are associated immune-mediated adverse events (ir-AEs) which can involve multiple organs. Cardiovascular manifestations include accelerated atherosclerosis [2], and myocarditis which has an estimated incidence of 1–2% [3] but reported fatality rate of 25 to 40% [4, 5]. Increasingly, milder elevation of troponin levels has been associated the use of ICIs [6, 7]. Given the severity of this complication and impact on downstream therapeutic decisions, timely diagnosis is essential in order to guide subsequent clinical management.

Recently published guidelines recommend the diagnosis of ICI myocarditis with pathohistological or clinical methods [8]. Cardiac imaging in the form of echocardiography and cardiac magnetic resonance imaging (CMR) are the recommended imaging modalities in the diagnosis of ICI-associated myocarditis [8]. However, clinical studies have demonstrated intermediate diagnostic performance of CMR in the diagnosis of ICI myocarditis using the 2018 modified Lake Louise criteria [9, 10]. ^18^F-fluorodeoxyglucose (FDG) positron emission tomography (PET) imaging is a highly sensitive imaging test for the detection of inflammatory heart disease such as sarcoidosis and myocarditis [11, 12]. ^18^F-FDG PET imaging has been used in the diagnosis of ICI myocarditis with varying results [13], and could identify patients with early stage disease in case reports [14, 15]. Current international guidelines do not recommend PET in the diagnostic workup [8, 16].

Data on both CMR with ^18^F-FDG PET imaging in the diagnosis of ICI myocarditis is lacking. The purpose of this study was to investigate the role of both modalities in the diagnosis of ICI myocarditis, and determine diagnostic agreement between CMR and ^18^F-FDG PET imaging in patients with suspected ICI myocarditis.

## Methods

### Study design and population

In this retrospective, single-centre observational cohort study at Royal Brompton Hospital, cancer patients with suspected ICI myocarditis, and had partially met the 2022 ESC Cardio-Oncology Guidelines’ [8] definition of ICI myocarditis, were enrolled. Patients were included in the study if they were 18 years old and above, were currently receiving ICI therapy for the treatment for cancer, and had both CMR and ^18^F-FDG PET imaging performed in the diagnostic process of ICI myocarditis between January 2017 and April 2023. The study was approved by the institutional review board at Guy’s and St Thomas Hospital National Health Service Foundation Trust and the United Kingdom Health Research Authority.

### Definitions and data collection

Patients were identified if they met the inclusion criteria. Patients’ demographics including age and gender, and modifiable cardiovascular risk factors were captured. Cancer-specific variables such as type of primary malignancy and ICI received, and date of commencement were included. Corresponding clinical data including cardiovascular (CV) symptoms, cardiac biomarkers, electrocardiogram (ECG) readings were obtained to accurately phenotype the patients.

### Imaging

#### CMR

CMR imaging was performed on a 1.5T or 3T magnet including ECG gating, breath-holding, using recommended guidelines [17]. Following localising scans, long axis 2, 3 and 4 chamber and short axis images of the left and right ventricle were obtained. Exam protocols included cine balanced steady state free precession imaging for left ventricular functional and mass assessment and T2-weighted imaging employing either T2 short tau inversion recovery or spectral attenuated inversion recovery techniques. Pre-contrast T1 and T2 maps were performed, and T1 and T2 values were measured. Late gadolinium enhancement (LGE) images were performed 10 to 15 min after a gadolinium-based contrast agent. The CMR images and data were interpreted by experienced cardiologists who was accredited by the SCMR and/or EACVI and reviewed again by an independent reader. CMR findings were reported in line with recommendations for reporting CMR scans [18].

### ^18^F-FDG PET

Patients were instructed to take low-carbohydrate, high-fat, high-protein diet for 24 h followed by a minimum of 6 h fasting before PET scan examinations according to previously published cardiac FDG-PET/CT guidelines [19]. 250 MBq of FDG was administered intravenously and imaging was acquired 60–90 min following injection. The scans were performed on an integrated whole-body PET system (GE Discovery ST 4, GE Healthcare, Amersham, UK) and 3D list mode data was acquired with ECG gating as a dedicated Cardiac PET-CT scan to assess for myocardial inflammation. A low-dose CT was acquired for attenuation correction. Standardized uptake values (SUV) were obtained in the regions were obtained. The images were read by an experienced radiologist, and reviewed again by an independent radiologist who was blinded to the clinical data of the patients.

### Statistical analysis

Continuous data were tested for normality with the Shapiro-Wilk test. Normally distributed continuous data are presented as mean ± standard deviation (SD) while non-normally distributed continuous data are presented as median. A *P*-value < 0.05 was considered significant. Analyses were performed with IBM SPSS Statistics for Windows, version 23.0 (IBM Corp., Armonk, N.Y., USA).

## Results

### Patient characteristics

Twelve patients who fulfilled the inclusion criteria were identified from the centre’s Cardio-Oncology registry. This cohort had a mean age of 60 *±* 15 years old and 58% were male (Table 1). All patients had metastatic disease with a range of primary malignancies including melanoma (*n* = 2), urological (*n* = 2), colorectal (*n* = 2), and renal, breast, gynaecological, lung, head and neck cancers, and sarcoma (1 case each) (Table 1). All patients were receiving either single or dual ICIs when ICI myocarditis was suspected. The most common ICI were Programmed Cell Death Protein-1 (PD-1) inhibitors such as Durvalumab, Nivolumab and Pembrolizumab (*n* = 9). Cytotoxic T lymphocyte associated protein-4 (CTLA-4) inhibitor Ipilimumab (*n* = 5) and Programmed cell death ligand-1 (PD-L1) (Enfavolimab and Atezolizumab (*n* = 3)), and Relatimab, a Lymphocyte activation gene-3 (LAG-3) inhibitor (*n* = 1). Combination ICI with Nivolumab and Ipilimumab were used in 4 instances (Table 1). The median number of days between CMR and PET imaging was 10 (6.5–42.5) days. Corticosteroid therapy was not initiated before CMR or PET imaging and were only initiated after both scans were performed.

**Table 1:** Patient characteristics

Variable	Patients with suspected ICI myocarditis (*n* = 12)
**Age (years)**	60 ± 15
**Gender**	
Male	7 (58%)
Female	5 (42%)
**Cardiovascular Risk Factors**	
Hypertension	4 (33%)
Hyperlipidemia	3 (25%)
Diabetes Mellitus	1 (8%)
Smoking (Current/Ex)	4 (33%)
**Cancer Type**	
Gynecological	1 (8%)
Head & neck	1 (8%)
Lung	1 (8%)
Breast	1 (8%)
Sarcoma	1 (8%)
Urological	2 (17%)
Melanoma	2 (17%)
Colorectal	2 (17%)
Renal	1 (8%)
**Immune Checkpoint Inhibitor Type**	
PD-1	9 (75%)
CTLA-4	5 (42%)
PD-L1	3 (25%)
LAG-3	1 (8%)
CTLA-4 + LAG 3	1 (8%)
PD-1 + CTLA-4	4 (33%)
**Cardiovascular (CV) symptoms**	
Asymptomatic	6 (50%)
Biomarker Elevation	4 (33%)
Pericardial effusion	1 (8%)
Left ventricular ejection fraction decline	1 (8%)
Symptomatic	6 (50%)
Heart failure	3 (25%)
Arrhythmias	2 (17%)
Recurrent syncope	1 (8%)
**Biomarkers**	
Patients with elevated Troponin I or hs-Troponin I	5 (42%)
Troponin I level (ng/L)	17 *±* 7
hs-Troponin I level (ng/L)	41 *±* 52
Patients with elevated BNP or NT-proBNP	12 (100%)
BNP (ng/L)	122 *±* 96
NT-proBNP (ng/L)	11,125
Patients with elevated Creatine Kinase (CK)	1 (8%)
CK (IU/L)	723
**ECG findings**	
Normal sinus rhythm	5 (42%)
Tachycardia	3 (25%)
T wave inversions	2 (17%)
Premature ventricular complexes	1 (8%)
Paced rhythm	1 (8%)

### Cardiovascular (CV) symptoms, electrocardiogram (ECG) and blood test results

All of the patients in this study were clinically stable and did not require emergent treatment. Half of the patients (*n* = 6) were asymptomatic on presentation. Three of these patients had asymptomatic elevation of cardiac biomarkers, 1 patient had new onset pericardial effusion on echocardiogram, and 1 other patient had an incidental decline of left ventricular ejection fraction on echocardiogram. Amongst those with symptoms, 3 patients presented with heart failure symptoms, 2 patients had arrhythmias, and 1 had recurrent syncope. Three of the cases had abnormal ECG findings such as premature ventricular complexes and/or new T-wave inversion. Three (25%) had sinus tachycardia. More than one third of the cases had an elevated high-sensitive troponin I (hs-Trop I) or Troponin I levels. The mean hs-Trop I was 41 *±* 52ng/L and the mean Troponin I level was 17 *±* 7ng/L (Table 1). Coronary cause for troponin elevation was excluded with the use of computed tomography imaging of the coronary arteries (CTCA) in 2 out of the 5 patients with elevated troponins, and there was no evidence of acute coronary syndrome in these cases. All of the cases had an elevated B-type Natriuretic Peptide (BNP) or N-terminal pro B-type natriuretic peptide (NT-proBNP) level. The mean BNP was 122 *±* 96ng/L (Table 1).

Amongst those without an elevated troponin (*n* = 7), there was a suspicion of ICI myocarditis in 4 of the patients who had CV symptoms (palpitations, syncope and heart failure) while receiving ICIs. The remaining 3 patients were asymptomatic but had a decline of LVEF, development of pericardial effusion, or isolated elevated BNP levels, thus prompting the managing physicians to suspect ICI myocarditis (Table 1). Details of each patient’s CV symptoms are listed in greater detail under the Supplementary Material section.

### CMR findings

The mean indexed left ventricular end diastolic volume (LVEDV), left ventricular end systolic volume (LVESV), mass, and left ventricular ejection fraction (LVEF) were 86 *±* 21ml/m^2^, 42 *±* 20ml/m^2^, 76 *±* 22 g/m^2^ and 56 *±* 11% respectively. The mean right ventricular end diastolic volume (RVEDV), right ventricular end systolic volume (RVESV), right ventricular ejection fraction (RVEF) were 83 *±* 20ml/m^2^, 38 *±* 17ml/m^2^ and 58 *±* 9%, respectively. Three (25%) of the patients had myocardial oedema demonstrated on T2-STIR imaging. Trivial to moderate pericardial effusion was seen in 5 of the patients. In 1.5 Tesla (T) studies, 5 patients had an elevated native T1 while 3 patients had an elevated native T2. The mean native T1 was slight elevated at 1074 *±* 44ms (normal 975-1065ms), and T2 values were at the upper limit of normal with 54 *±* 5ms (normal < 55ms). There was a significant difference in myocardial T1 values between CMR negative and positive cases (1065 *±* 19ms vs. 1086 *±* 19ms, *p* = 0.03). However, no significant difference was seen between the 2 groups for T2 values (52 *±* 5ms vs. 56 *±* 4ms, *p* = 0.87). Late gadolinium enhancement (LGE) was seen in 8 (67%) of the cases. The LGE pattern was seen in the mid-wall (4/8), followed by subendocardial (3/8) and subepicardial (1/8) (Table 2).

**Table 2:** Imaging findings

Variable	Patients with suspected ICI myocarditis (*n* = 12)		
CMR			
Left Ventricle values			
Indexed EDV (ml/m^2^)	86 *±* 21		
Indexed ESV (ml/m^2^)	42 *±* 20		
EF (%)	56 *±* 11		
Mass index (g/m^2^)	76 *±* 22		
Right Ventricle values			
Indexed EDV (ml/m^2^)	83 *±* 20		
Indexed ESV (ml/m^2^)	38 *±* 17		
EF (%)	58 *±* 9		
Myocardial oedema by T2-STIR	3 (25%)		
Pericardial effusion			
None	7 (58%)		
Trivial	2 (17%)		
Mild	2 (17%)		
Moderate	1 (8%)		
Severe	0 (0%)		
Number of patients with			
Elevated native T1	5 (42%)		
Elevated native T2	3 (25%)		
Myocardial T1 and T2 values (ms) in 1.5T studies			
Native T1 (ms)	1074 *±* 45		
Native T2 (ms)	54 *±* 5		
Comparison of T1 and T2 values (ms) amongst CMR negative and positive studies			
	CMR -	CMR +	*p* value
T1 (ms)	1065 *±* 59	1086 *±* 19	0.03
T2 (ms)	52 + 5	56 + 4	0.87
Late Gadolinium Enhancement	8 (67%)		
Mid-wall	4 (50%)		
Subendocardial	3 (38%)		
Subepicardial	1 (13%)		
^**18**^ **F-FDG PET/CT**			
Positive studies	4 (33%)		
Mean maximum SUV	3.5 *±* 1.7		

### PET-CT findings

In all of the cases, there was sufficient myocardial suppression following dietary preparation. Four (33%) out of the 12 patients had ^18^F-FDG PET findings positive for myocardial inflammation. The mean standard uptake value (SUV) amongst these positive cases was 3.5 *±* 1.7 (Table 2).

### CMR and PET-CT comparison

There was agreement between CMR and ^18^F-FDG PET findings in 7 patients, where 6 patients did not meet the 2018 Lake Louise criteria for myocarditis and were also negative for myocardial inflammation on ^18^F-FDG PET imaging. One patient had positive CMR and ^18^F-FDG PET findings for myocardial inflammation. Discordance between the two imaging techniques were seen in 5 patients: 3 cases did not meet the CMR Lake Louise criteria for myocarditis but were positive for myocardial inflammation on ^18^F-FDG PET; 2 cases met the CMR Lake Louise criteria but the respective ^18^F-FDG PET scans did not demonstrate inflammation (Table 3).

**Table 3:** Agreement between CMR and ^18^F-FDG PET studies

	^18^F-FDG PET		Total
	+ Myocarditis	- Myocarditis	
**CMR**			
+ Myocarditis	1	2	3
- Myocarditis	3	6	9
**Total**	4	8	12

In concordant positive study (*n* = 1), the mean T1 and T2 values were elevated at 1102ms and 58ms respectively. Myocardial oedema was demonstrated on T2-STIR imaging and LGE was present. The maximum SUV on ^18^F-FDG PET was 2.4 (Table 4). Amongst those with concordant negative studies, the mean T1 and T2 values were 1114 *±* 23ms and 50ms respectively. There was no myocardial oedema demonstrated on T2-STIR sequence, although LGE changes were identified in all 6 studies (Table 4). Discordant studies which were positive for myocarditis in CMR, but negative on ^18^F-FDG PET, had mean T1 and T2 values were 1078 *±* 18ms and 55 *±* 6ms respectively. Myocardial oedema on T2-STIR sequence and LGE were present in 1 case each (Table 3). Discordant studies (*n* = 3) which were negative for myocarditis in CMR but positive on ^18^F-FDG PET, had mean T1 and T2 valves of 1017 *±* 24ms and 54 *±* 8ms respectively. The mean maximal SUV in these cases was 6.2 *±* 6.1. Myocardial oedema on T2-STIR sequence and LGE was detected in in 1 case each (Table 4).

**Table 4:** Clinical and imaging findings in concordant and discordant studies

Variables	Concordant Studies		Discordant Studies	
	CMR and PET positive (*n* = 1)	CMR and PET negative (*n*-6)	CMR positive and PET negative (*n* = 2)	CMR negative and PET positive (*n* = 3)
**Clinical**				
Number of patients on dual ICI therapy	0	5	0	0
**CMR**				
Left Ventricle values				
Indexed EDV (ml/m^2^)	117	92 *±* 39	68 *±* 4	75 *±* 19
Indexed ESV (ml/m^2^)	67	46 *±* 20	24 *±* 10	40 *±* 22
EF (%)	56	52 *±* 12	71 *±* 8	58 *±* 2
Right Ventricle values				
Indexed EDV (ml/m^2^)	158	88 *±* 27	82 *±* 25	72 *±* 1
Indexed ESV (ml/m^2^)	95	39 *±* 20	35 *±* 5	28 *±* 8
EF (%)	49	58 *±* 10	57	61 *±* 10
Positive myocardial oedema by T2-STIR	1	0	1	1
Pericardial effusion	1 trivial	2 mild	0	1 trivial 1 moderate
Myocardial T1 and T2 values (ms) in 1.5T studies				
Native T1 (ms)	1102	1114 *±* 23	1078 *±* 18	1017 *±* 24
Native T2 (ms)	58	50	55 *±* 6	54 *±* 8
Positive Late Gadolinium Enhancement	1	5	1	1
^**18**^ **F-FDG PET**				
Positive studies	1	0	0	3
Mean maximum SUV	2.4	0	0	6.2 *±* 6.1

### Impact of imaging results on management

ICIs were stopped in patients who demonstrated myocardial inflammation on ^18^F-FDG PET imaging. They were also treated with intravenous and subsequently oral steroids. Amongst those with negative studies on ^8^F-FDG PET imaging, they were deemed not to have ICI myocarditis and did not receive steroidal treatment. This group of patients also continued on their cancer immunotherapy.

## Discussion

Cardiovascular immune related adverse events are uncommon but carry significant mortality and morbidity [20]. Diagnosis of myocarditis can be made through detection of early tissue responses in the form of myocardial oedema, vasodilatation and myocyte necrosis, and replacement fibrosis later on [21]. Endomyocardial biopsy (EMB) has been recommended as a gold standard in diagnosis with its ability to provide histopathological diagnosis [22]. While confirmatory when positive, EMB has a sensitivity of 45% for the diagnosis of myocarditis [23], and EMB is an invasive test with a small risk of serious complications [24].

In this study, we found important differences in CMR and PET for the assessment of patients with ICI myocarditis. One important aspect to consider for the interpretation of these findings is that CMR and PET evaluate myocardial inflammation in different approaches. CMR can be used to assess for myocardial oedema as a surrogate from inflammation, whereas PET assesses myocardial inflammation as a result of increased metabolic activity from cardiomyocytes with active inflammation.

CMR may be helpful in the diagnosis and monitoring of cardiovascular damage in cancer patients [25], and for the diagnosis of myocarditis or monitoring for disease progression. In the European of Society (ESC) 2022 Cardio-Oncology guidelines, CMR and echocardiogram had a Class I recommendation for the diagnosis of ICI myocarditis, and the 2018 modified Lake Louise criteria was a major criterion for its diagnosis in the guideline [8]. The strength of evidence for myocarditis was increased if myocardial oedema was present with markers of inflammatory myocardial injury on the CMR study [8, 26]. The 2018 modified LL criteria outperformed the original criteria in the diagnosis of acute myocarditis with a significant improvement of sensitivity and specificity (88% and 96%) respectively [27]. These criteria require both the presence myocardial oedema seen on T2-weighted imaging, and that of fibrosis in T1, extracellular volume (ECV) and LGE imaging [28].

However, CMR findings in ICI myocarditis can be variable and less predictable [29]. There was lower rate of late gadolinium enhancement (LGE) and lower sensitivity of the Lake Louise criteria in ICI myocarditis than viral myocarditis [30]. In an international registry of patients with ICI myocarditis, elevated T2-weighted short tau inversion recovery (STIR) was only present in 28%, LGE was seen in 48% of patients, while 42% of the cases did not even have an abnormal T2-STIR or LGE [9]. Timing of when CMR was performed was also crucial to diagnosis. It was demonstrated in the same study that LGE increased from 22 to 72% (*P* < 0.001) if the CMR was performed beyond day 4 of the onset of symptoms [9]. Myocardial characterisation with the use of parametric mapping has been found to aid in the diagnosis of ICI myocarditis. In another study of 136 patients with ICI myocarditis, abnormal T1 and T2 values were seen in 78% and 43% respectively, and T1 mapping had an impact on prognosis [10]. However, T1 mapping is non-specific and can be elevated by a variety of cardiac conditions that result in changes in the myocardial architecture [31].

^18^F-FDG PET is increasingly used in the evaluation of myocarditis. Although its use is not recommended in the current guidelines for cardio-oncology [8], some studies have shown promising results in diagnosing other types of myocarditis. In one study, the sensitivity and specificity of PET was 74% and 97% compared to CMR [11]. ^18^F-FDG PET could be considered as an alternative non-invasive imaging modality in stable patients with contraindications to CMR or in those with suspected concomitant systemic autoimmune disease [32]. However, ^18^F-FDG PET imaging’s performance in ICI myocarditis is limited. In a study of 31 patients with treated suspected ICI myocarditis who underwent ^18^F-FDG PET studies, there was low sensitivity and negative predictive value demonstrated, although the majority of patients had already been initiated on corticosteroid therapy which may have inadvertently blunted the FDG signal [13]. Attempts have been made with the use of novel tracers in PET studies to identify early ICI myocarditis. ^68^Ga-DOTA(0)-Phe(1)-Tyr(3)-octreotide (^68^Ga-DOTATOC) [33] and 68Ga-FAPI PET-CT [34] have been studied in small studies and shown to be useful in detecting inflammation in early stages of ICI myocarditis. Larger studies need to be performed to understand the clinical application in this this cohort of patients.

To our knowledge this is the first study to compare CMR and PET imaging for the diagnosis of ICI myocarditis. Whilst there was agreement in several cases, this study provides a signal that both CMR and ^18^F-FDG PET-CT provide complementary information for the non-invasive diagnosis of ICI myocarditis. CMR allows for accurate assessment of morphology, function, and tissue characterisation by providing information on myocardial oedema and fibrosis [35]. Whereas PET-CT may provide quantitative assessment of myocardial inflammation. Combining the strength of both modalities may provide complementary clinical information in challenging cases. Future studies should consider hybrid PET-CMR imaging, which can provide complementary information in just a single scan, reduce the number of imaging studies needed, and allow for quicker diagnosis and treatment for patient in order to guide subsequent clinical management.

### Limitations

The authors acknowledge the limitations of a small sample size, in a retrospective, single-centre cohort study. The small sample size reflects the rarity of myocarditis as a complication of ICI. We also note that endomyocardial biopsy was not performed in all of the cases. However, sampling errors can occur with use of EMB. An EMB is also an invasive procedure with the potential for complications. The benefits of an EMB in the diagnosis of ICI myocarditis in this cohort of largely asymptomatic patients with mild troponin elevation are questionable. Thirdly, the CMR protocols used in this cohort were heterogeneous. We agree that a standardised CMR protocol would have improved the accuracy in diagnosing myocarditis. Furthermore, since the patients underwent two distinct imaging tests, it is likely that there were some potential diagnostic challenges which may have introduced selection bias of the difficult cases into this study. Finally, the CMR and PET scans were not performed on the same day, which may have introduced biological variability into the findings presented.

## Conclusion

Diagnosing ICI myocarditis is challenging, especially in cases with mild symptoms and biomarker elevation. Non-invasive imaging modalities are increasingly used in diagnosis. This is the first study to describe CMR and ^18^F-FDG-PET in suspected ICI myocarditis, and it demonstrates the presence of some agreement between both modalities. This suggests that CMR and PET provide complementary clinical information in the diagnostic process. Larger studies will be required to test this hypothesis further, and also evaluate the role of hybrid PET-CMR imaging in the diagnosis of ICI myocarditis.

### Electronic supplementary material

Below is the link to the electronic supplementary material.

Supplementary Material 1

## Data Availability

No datasets were generated or analysed during the current study.

## Abbreviations

CMR: Cardiac magnetic resonance
CTLA-4: Cytotoxic T lymphocyte associated protein-4
EMB: Endomyocardial biopsy
Hs-Trop I: High-sensitive troponin I
ICI: Immune checkpoint inhibitors
Ir-AE: Immune related adverse event
LAG-3: Lymphocyte activation gene-3
PD-1: Programmed Cell Death Protein-1
PD-L1: Programmed Cell Death ligand-1
^18^F-FDG PET: ^18^F-fluorodeoxyglucose positron emission tomography

## Declarations

### Disclaimer

The views expressed in this article are the authors’ own and not an official position of Royal Brompton Hospital.
