# Supplementary material for: Complementary use of cardiac magnetic resonance and 18 F-FDG positron emission tomography imaging in suspected immune checkpoint inhibitor myocarditis
Source: Cardiooncology. 2024 Aug 22;10:53. doi: 10.1186/s40959-024-00250-0 (PMC11340117; doi:10.1186/s40959-024-00250-0)
Supplement: Supplementary file 1 — Supplementary Material 1 [file 40959_2024_250_MOESM1_ESM.docx]

**APPENDIX**

**Appendix 1. Patient characteristics and symptoms**

| **Patient** | **Gender** | **Age (years old)** | **Malignancy** | **Type of ICI** | **Cardiac Symptoms** |
| --- | --- | --- | --- | --- | --- |
| 1 | Female | 73 | Ovarian cancer | Durvalumab | Shortness of breath and palpitations while receiving Durvalumab. Holter showed runs of premature ventricular complexes. Troponin was negative but BNP was very slightly elevated. |
| 2 | Male | 46 | Metastatic squamous cell cancer | Nivolumab | Recurrent syncope with preceding dizziness while receiving Durvalumab. Troponin was negative while BNP was very mildly elevated. |
| 3 | Male | 62 | Metastatic renal cell cancer | Ipilimumab, Nivolumab | Previous anthracycline induced CTRCD . Developed chest pain, shortness of breath with new T wave inversions after receiving his 3^rd^ cycle of immunotherapy. Troponin was negative but NT-proBNP was significantly elevated. |
| 4 | Male | 60 | Metastatic melanoma | Ipilimumab, Nivolumab  Pembrolizumab | Shortness of breath and palpitations on exertion while receiving Pembrolizumab. . Troponin was negative but BNP was mildly elevated. |
| 5 | Male | 75 | Metastatic melanoma | Ipilimumab, Nivolumab | Worsening shortness of breath and lower limb oedema. BNP and troponin I were elevated. |
| 6 | Female | 59 | Adenocarcinoma of lung | Ateolizumumab | Asymptomatic troponin I elevation. BNP was slightly elevated. |
| 7 | Male | 55 | Metastatic ureteric cancer | Ipilimumab, Nivolumab | Shortness of breath on exertion with lower limb oedema. Troponin I was not elevated while BNP is mildly elevated. |
| 8 | Female | 78 | Metastatic bladder cancer | Pembrolizumab | Asymptomatic rise in BNP while troponin was negative |
| 9 | Male | 71 | Metastatic colorectal adenocarcinoma | Nivolumab, Relatlimab | Shortness of breath with declined in LVEF on echocardiogram. Moderate sized pericardial effusion on echocardiogram. Troponin I was normal. |
| 10 | Male | 47 | Metastatic sarcoma | Envafolimab | Asymptomatic rise in Troponin I and BNP levels. New changes noted on ECG too. |
| 11 | Female | 27 | Metastatic colorectal adenocarcinoma | Pembrolizimab | Palpitations with declined in LVEF seen on echocardiogram. Both troponin I and BNP are mildly elevated. |
| 12 | Female | 61 | Metastatic breast cancer | Ateolizumumab | Asymptomatic rise in Troponin I and DNP. New left bundle branch block noted on ECG and pericardial effusion on echocardiogram. |
